# Heterogeneity in Neutrophil Microparticles Reveals Distinct Proteome and Functional Properties*[Fn FN2]

**DOI:** 10.1074/mcp.M113.028589

**Published:** 2013-05-08

**Authors:** Jesmond Dalli, Trinidad Montero-Melendez, Lucy V Norling, Xiaoke Yin, Charles Hinds, Dorian Haskard, Manuel Mayr, Mauro Perretti

**Affiliations:** From the ‡The William Harvey Research Institute, Barts and The London School of Medical, Charterhouse Square, London EC1M 6BQ, United Kingdom;; §Cardiovascular Division, The James Black Centre, King's College, University of London,125 Coldharbour Lane, London SE5 9NU, United Kingdom;; ¶Department of Intensive Care, Barts Health NHS Trust, London, United Kingdom;; ‖Department of Rheumatology and Department of Haematology, Imperial College Healthcare NHS Trust, London, United Kingdom

## Abstract

Altered plasma neutrophil microparticle levels have recently been implicated in a number of vascular and inflammatory diseases, yet our understanding of their actions is very limited. Herein, we investigate the proteome of neutrophil microparticles in order to shed light on their biological actions. Stimulation of human neutrophils, either in suspension or adherent to an endothelial monolayer, led to the production of microparticles containing >400 distinct proteins with only 223 being shared by the two subsets. For instance, postadherent microparticles were enriched in alpha-2 macroglobulin and ceruloplasmin, whereas microparticles produced by neutrophils in suspension were abundant in heat shock 70 kDa protein 1. Annexin A1 and lactotransferrin were expressed in both microparticle subsets. We next determined relative abundance of these proteins in three types of human microparticle samples: healthy volunteer plasma, plasma of septic patients and skin blister exudates finding that these proteins were differentially expressed on neutrophil microparticles from these samples reflecting in part the expression profiles we found *in vitro*. Functional assessment of the neutrophil microparticles subsets demonstrated that in response to direct stimulation neutrophil microparticles produced reactive oxygen species and leukotriene B_4_ as well as locomoted toward a chemotactic gradient. Finally, we investigated the actions of the two neutrophil microparticles subsets described herein on target cell responses. Microarray analysis with human primary endothelial cells incubated with either microparticle subset revealed a discrete modulation of endothelial cell gene expression profile. These findings demonstrate that neutrophil microparticles are heterogenous and can deliver packaged information propagating the activation status of the parent cell, potentially exerting novel and fundamental roles both under homeostatic and disease conditions.

The emerging notion that cells can communicate by packaged information represents a major shift in our understanding of cell-to-cell interaction in complex settings including inflammation (1). Packaging of mediators (irrespective of their chemical nature) in structures that can be transported through the vascular and lymphatic systems might avoid their rapid dilution and removal by biological fluids and allow the target cell or tissue to receive a biologically relevant amount of a given molecule. As an example, TNF-α produced by mast cells in the mouse paw can reach the lymph nodes unmodified, wrapped up in small structures or vesicles (2). In this respect, the last few years have witnessed augmented understanding in microparticle function.

Described over 50 years ago (reviewed in (3, 4), microparticles are heterogeneous in nature with their size varying between 0.2 and 1.0 μm, and are characterized by an outer membrane composed of a phospholipid bilayer and cell surface proteins. The mechanism of microparticle production is not fully understood, though it may follow processes not dissimilar from those observed in apoptosis, involving membrane detachment from the anchoring cytoskeleton and loss of membrane symmetry, which leads to exposure of negatively charged phospholipids (5–7). Proteins found on the outer leaflet of the microparticle cell membrane are believed to reflect both the origin and activation status of the parental cell (8, 9); for instance, microparticles from neutrophils express CD66b and CD62L (10, 11). We have recently identified the selective expression of the potent anti-inflammatory and proresolving protein Annexin A1 (ANXA1) on the surface of microparticles generated from neutrophils adherent to endothelial monolayers, when compared with those prepared from quiescent neutrophils (12). Microparticle production is not restricted to one subset of cells and using cell specific antigens the relative contribution of different cell types to the total microparticle population in a particular environment can be assessed. This has allowed for the analysis of different microparticle populations (the focus being by and large platelet- and endothelial-derived microparticles) in a number of pathologies in the quest to identify robust biomarkers for disease and treatment (13–15). With regard to inflammatory diseases, examples would include plasma samples in sepsis (16), psoriatic arthritis (17), and scleroderma (18). However, the vast majority of these studies have only determined microparticle expression patterns with respect to the cell type of origin, without addressing the possibility that microparticle composition—even when generated from the same leukocyte subset—might differ in relation to disease status and/or mode of cell activation. Of note recent work has also demonstrated that the production of neutrophil microparticles during self-limited inflammation is temporally regulated suggesting that these microparticles are important in orchestrating inflammation-resolution (1).

Recent work has established that microparticles can elicit a variety of biological processes ranging from angiogenesis to anti-inflammation; so that it is very unlikely they can continue to be considered “cell debris,” as initially postulated. The following are some examples, relevant to the present study. Ingestion of platelet microparticles alters the phenotype of macrophages, leading to the false identification of endothelial cell progenitor cells in culture (19). Likewise, sonic hedgehog can be transferred, *via* microparticles, to dysfunctional endothelial cells, restoring the activity of nitric oxide synthase with downstream production of nitric oxide (20). Microparticles can carry functionally active receptor proteins to target cells (21, 22). Finally, *in vivo* generation of microparticles has been observed within the inflamed microcirculation. Real time analysis of leukocyte recruitment has visualized microparticle release from leukocytes squeezing through an endothelial barrier, providing evidence for their formation *in vivo* together with potential functional relevance in relation to cell migration (23).

On stimulation, neutrophils produce microparticles with rapid and nongenomic anti-inflammatory properties, *in vitro* and *in vivo*, reliant on their expression of ANXA1 (12). Whereas these findings are consistent with those obtained by Gasser and colleagues (24) who described inhibitory properties of neutrophil microparticles, other studies have suggested that the same cell type can produce microparticles that elicit activating properties, for instance upon incubation with endothelial cells or monocytes for longer time-points (25, 26). Thus, to gain further insight into the potential mechanisms involved in mediating such distinct effects, we deemed it important to determine the total proteome of neutrophil microparticles. Having established that different stimulation conditions yield microparticle populations with distinct protein profiles, we corroborated our observations in two distinct clinical scenarios, characterizing neutrophil microparticles from skin blister exudates and plasma samples from sepsis patients using a select group of proteins identified in our proteomic profile. We also established that the two microparticles subsets differentially modulate endothelial cell gene expression profile and thereby function, as determined by connectivity map analysis.

## EXPERIMENTAL PROCEDURES

Unless otherwise specified, materials were obtained from Sigma-Aldrich Ltd (Poole, UK). Human cells were prepared according to a protocol approved by the local Research Ethics Committee (P/00/029 ELCHA). Plasma samples were obtained from patients suffering from severe sepsis/septic shock (defined according to The American College of Chest Physicians/Society of Critical Care Medicine consensus definitions) caused by community-acquired pneumonia (CAP) using a protocol approved by the Multicenter Research Ethics Committee (08/H0505/78).

### 

#### 

##### Microparticle Generation and Characterization

##### Generation of Different Samples of Microparticles

Human neutrophil microparticles were prepared from peripheral blood neutrophils obtained from healthy volunteers as previously described (12). Two methods of neutrophil microparticle production were employed. In the first case neutrophils were resuspended in RPMI 1640 (Invitrogen, Paisley, UK) at a concentration of 2 × 10^7^ cells/ml incubated for 20 min at 37 °C and then stimulated for a further 20 min at 37 °C with 1 μm fMLF: this subset of microparticles will be referred to, hereafter, as *Fl*uid *P*hase or FlP^1^ microparticles. The second protocol involved pre-incubation of neutrophils, resuspended at 2 × 10^7^ cells/ml, over a HUVEC monolayer for 20 min at 37 °C before addition of fMLF (1 μm; 20 min at 37 °C): this subset of microparticles will be referred to *Im*mobilized *P*hase or ImP microparticles. All incubation assays were conducted in the absence of fetal calf serum and, for the ImP microparticles. HUVEC monolayers were washed with PBS before addition of the neutrophils. In both cases, cell supernatants were collected and cells removed by two successive centrifugations at 3000 × *g* for 10 min at 4 °C, before pelleting the microparticles by centrifugation at 100,000 × *g* for 1 h at 4 °C, as described (12). Microparticle pellets were washed with Dulbecco phosphate buffered solution (DPBS), resuspended and stored at −80 °C before further analysis.

Blood (4 ml) from healthy volunteers or septic patients was centrifuged at 4 °C for 10 min at 1600 × *g*, to produce the plasma which was pipetted into cryovials and stored at −80 °C, before centrifugation and subsequent ultracentrifugation, as describe above. For the septic patients plasma was obtained on the first day of admission to the Intensive Care Unit.

Exudate microparticles were prepared from skin blisters, generated by application of 0.1% cantharidin as previously described (27), and were harvested at the 24 h time point, because it corresponds to intense neutrophilic response (28, 29). Table II reports the demographics of these volunteers.

##### Proteomic Analysis

Microparticle extracts from two different preparations of each group were reduced in Laemmli sample buffer as described (12) and run in duplicate (four analyses for each set of microparticles). After separation by 5–20% tris-glycine polyacrylamide gel electrophoresis and silver-staining (PlusOne Silver staining kit, GE Healthcare), gel bands were subjected to in-gel digestion with trypsin using an Investigator ProGest (DIGILab) robotic digestion system. Tryptic peptides from the digests were separated on a nanoflow LC system (UltiMate3000, Thermo Fisher Scientific) and eluted with a 40 min gradient (10–25% B in 35 min, —40% B in 5 min, 90% B in 10 min and 2% B in 30min where A = 2% acetonitrile, 0.1% formic acid in high performance liquid chromatography (HPLC) (H_2_O and B = 90% acetonitrile, 0.1% formic acid in HPLC H_2_O). The column (PepMap100 C18, 25-cm length, 75-μm internal diameter, 3-μm particle size, Thermo Fisher Scientific) was coupled to a nanospray source (Picoview) using RePlay (Advion) (30). Spectra were collected from a high-mass accuracy analyzer (LTQ Orbitrap XL, Thermo Fisher Scientific) using full MS scan mode over the mass-to-charge (*m*/*z*) range 450–1600. MS/MS was performed on the top six ions in each MS scan using the data-dependent acquisition mode with dynamic exclusion enabled. MS/MS peaklists were generated by extract_msn.exe and matched to human database (UniProtKB/Swiss-Prot Release 14.6, 20333 protein entries) using SEQUEST v.28 (rev. 13), (Bioworks Browser 3.3.1 SP1, Thermo Fisher Scientific) and X! Tandem, (Version 2007.01.01.2). Carboxyamidomethylation of cysteine was chosen as fixed modification and oxidation of methionine as variable modification. The mass tolerance was set at 50ppm for the precursor ions and at 1.0 AMU for fragment ions. Two missed cleavages were allowed. Scaffold (version 2.0.5, Proteome Software Inc., Portland, OR) was used to calculate the spectral counts and to validate MS/MS based peptide and protein identifications. According to the default values in the Scaffold software, the following peptide thresholds were applied; X! Tandem: -Log(Expect Scores) > 2.0, SEQUEST: deltaCn > 0.10 and XCorr > 2.5 (2+), 3.5 (3+) and 3.5 (4+). Peptide identifications were accepted if they could be established at greater than 95.0% probability as specified by the Peptide Prophet algorithm (31). Protein identifications were accepted if they could be established at greater than 99.0% probability (32) with at least two independent peptides and a mass accuracy of ≤10ppm of the precursor ion. Normalized spectral count for each proteins were used for quantitation and protein changes with Student *t* test *p* value < 0.05 (*n* = 4) were considered as significant.

##### Western Blot Analysis

Presence of a select group of proteins identified by proteomic analysis was confirmed through standard SDS-PAGE, loading extracts from ∼2 × 10^6^ microparticles per lane (Millipore, Watford, UK). Western blot was conducted with specific antibodies against AnxA1 (5 μg/ml; clone 1B), anti-Alpha-2-macroglobulin (A2MG; 5 μg/ml clone 3D1; Thermo Scientific, Hampshire, UK), anti-Ceruloplasmin (CERU; 5 μg/ml; clone 8; BD Biosciences, Oxford, UK), anti-Heat shock 70 kDa protein 1 (HSP71; 5 μg/ml; clone 4E7, AB Serotec, Oxford, UK), anti-Lactoferrin (TRFL; 5 μg/ml; clone L3262, Sigma-Aldrich, Poole, UK) or anti-β-actin (ACTB; 5 μg/ml; clone AC-74, Sigma-Aldrich) overnight at 4 °C followed by a 1 h incubation with either an HRP-conjugated goat anti-mouse IgG or goat anti-rabbit IgG (Dako, Cambridge, UK). Proteins were detected using an ECL detection reagent and visualized on Hyperfilm™ (GE Healthcare, Buckinghamshire, UK).

##### Flow-cytometric Analysis

To assess the homogeneity of the microparticle preparations, microparticles were suspended in PBS containing calcium and magnesium and incubated with either AnxAV (following manufacturer's instructions), mouse anti-human CD66b (clone: G10F5; BioLegend), CD14 (clone: M5E2, BD Biosciences), CD62P (clone: AK-4, BD Biosciences), CD41 (clone: HIP8, eBiosciences) or CD54 (clone: HCD54; Biolegend) fluorescently conjugated antibodies or relevant isotype controls for 20 min at room temperature and staining assessed using FACSCalibur or FACSCanto II flow cytometers and data analyzed using either CellQuestTM software (Becton Dickinson) or FlowJo (Treestar Inc).

To determine microparticle cell surface protein expression, a double-staining protocol was applied using an anti-CD66b PE conjugated antibody (1:25) and one of the following Alexa488 conjugated antibodies: anti-ANXA1 (1 μg/ml; Clone 1B), anti-A2MG (5 μg/ml; Clone 3D1; Thermo Scientific), anti-CERU (2 μg/ml; Clone 8; BD Bioscience), or anti-HSP71 (2 μg/ml; Clone 4E7; Ab Serotec). All these antibodies and relevant isotype controls were labeled in house using monoclonal antibody conjugation kits (Invitrogen, Paisley, UK; cat no: A20181) following manufacturer's instructions. In all cases, microparticles were incubated with the antibodies or relevant isotype controls for 45 min at 4 °C and before analysis with a FACSCalibur flow cytometer (Becton Dickinson, San Jose, CA) using CellQuestTM software (Becton Dickinson). Protein abundance was assessed by subtracting the mean fluorescent value for each of the proteins of interest from the value obtained for the relevant isotype control.

In separate experiments, protein levels on neutrophil microparticles in plasma obtained from healthy volunteers and septic patients, along with exudates obtained from cantharidin-elicited skin blisters, were tested using staining protocols outlined above.

##### Microparticle Functional Assays

##### Reactive Oxygen Species Determination

*Lucigenin assay*. Microparticles (∼6 × 10^5^) obtained from both FlP and ImP populations were pre-incubated in the presence of 5 μm Lucigenin for 10 min at 37 °C prior to incubation with PMA (16 μm; Merck Chemicals Ltd., Nottingham, UK) or vehicle, and luminescence was assessed in the thermostated chamber of a luminometer (Wallac VICTOR2 1420 Multilabel Counter, Perkin Elmer Life Science) for further 45 min at 37 °C. *DCFDA assay*. FlP and ImP microparticles were suspended at 2 × 10^5^ per ml in HBSS and incubated with or without pertussis toxin (1 μg/ml) for 3h at room temperature. These were then incubated with 10 μm DCFDA (30 min at 37 °C). A basal fluorescence reading in the FL1 channel of a FACSCalibur flow cytometer (Becton Dickinson) using CellQuestTM software (Becton Dickinson) was taken prior to stimulation with fMLF (1 μm) or vehicle.

##### Leukotriene (LT)B_4_ Generation

FlP or ImP microparticles (∼6 × 10^4^) were resuspended in Krebs Ringer bicarbonate buffered solution, prior to stimulation with 100 nm arachidonic acid (Calbiochem) with or without 10 nm A23187 (Sigma-Aldrich). After a further 15 min at 37 °C, LTB_4_ levels were measured using a Parameter™ LTB_4_ assay kit (R&D Systems, Abingdon, United Kingdom).

##### Chemotaxis

ImP or FlP microparticles were suspended at ∼3 × 10^7^ per ml in RMPI1640 that was filtered through a 0.2 μm filter (without FCS or supplements) and the extent of chemotaxis toward fMLF (1 μm) was assessed. Therefore, 27 μl of a solution of the chemotactic agents or a vehicle control were added to the bottom of a 96-well ChemoTx® plate equipped with 2 μm pore filters (Neuroprobe, Gaithersburg, USA) and the microparticle containing solution was added to the top. After 1h at 37 °C the remaining solution at the top of the chemotaxis chamber was removed and the membrane washed once with RPMI1640. Subsequently, the number of microparticles in the lower chamber was quantified using a previously calibrated flow cytometer as a function of the AnxAV positive events within the microparticle gate.

##### Microparticle Sensing by Endothelial Cells

##### Microarray Studies

HUVEC were grown to monolayers in six-well plates and co-incubated with buffer, FlP or ImP microparticles (∼8 × 10^5^) for 6h (at 37 °C and 5% CO_2_). RNA was subsequently extracted using an RNeasy® Plus mini kit (Qiagen, West Sussex, UK) following manufacturer's instructions. Three independently prepared HUVEC and microparticle preparations were employed for this analysis. RNA integrity was determined on RNA 6000 Nano LabChips (Agilent Technologies, Palo Alto, CA). Preparation of cRNA and hybridization on the whole genome microarrays HumanHT-12 v4 Expression BeadChip were performed according to the manufacturer's protocols using Custom Illumina® TotalPrepTM-96 Kit and Whole-Genome Gene Expression Direct Hybridization Assay (Illumina, Essex, UK). Raw measurements were processed by GenomeStudio software (Illumina). GenomeStudio checks that a probe has ≥3 beads present on the array (if not, the probe is considered to be missing), does a local background subtraction and condenses bead-level data into a single probe-level value per probe by removing outliers >3 median absolute deviations from the median, recalculating the mean of the remaining values. Data were quantile normalized and fold change expressed as the ratio treatment/control. Genes were considered to be significantly modulated if the mean signal obtained in either of the microparticle-treated groups was significantly higher (*p* < 0.05) than that obtained for the DPBS treated group. Unsupervised hierarchical clustering (heat map) was performed using Babelomics 4 (33). All microarray data are publicly available in the Gene Expression Omnibus (GEO) database with accession number GSE25154.

##### Quantitative Real-time PCR (qRT-PCR)

Results of the microarray data were validated by qRT-PCR. Real time-PCR was performed with 120 ng of cDNA per well, 1 μl primers and Power SYBR Green PCR Master Mix (Applied Biosystems, UK), using the ABI Prism 7900HT Sequence Detection System. Predesigned QuantiTect® Primer Assays (Qiagen) were used to measure the expression levels of: ATF3, CASP2, CCL3L3, CXCL5, HIF1A, IL1B, ICAM2, NLRP8, NOX4, NRG1, NRP1, PECAM1, PPT1, PTPRG, THBS1, and VCAM1, and absence of unspecific products was assessed by including a dissociation step. Gene expression values were calculated as log_2_(2^−ΔΔCt^) using GAPDH as endogenous control.

##### Functional Analysis

Functional analyses of identified proteins and the differentially expressed genes were performed using PHANTHER classification system v6.1 ((34)). The top 15 (for the proteomics analysis) or top 10 (gene analysis) pathways according to significance were selected.

##### In Silico Prediction of Drugs Connections

The two distinct gene expression signatures generated in this study (HUVEC cells treated either with ImP or FlP microparticles) were compared with the more than 7000 gene expression profiles obtained from 1309 small molecules contained in the current version (build 02) of the Connectivity Map (CMap) ((35)). The similarity between the gene expression profiles of interest and those contained in the CMap database is determined by the connectivity score, ranging from −1 to +1. Considering the hypothesis that if a drug has a gene expression signature that is similar to another drug could potentially be used to identify novel mechanisms of action, we focused on the analysis of the drugs showing a positive score. The top 10 drugs with the highest score were selected.

##### Statistical Analyses

Experiments were performed in triplicate and data are expressed as Mean ± S.E. Statistical differences were determined using one-way analysis of variance or Student's *t* test as appropriate, using GraphPad Prism™. A probability value less than 0.05 was taken as significant for rejection of the null hypothesis.

## RESULTS

### 

#### 

##### Neutrophil Microparticle Proteomic Characterization

##### ImP and FlP Neutrophil Microparticles

**Fig. 1.**
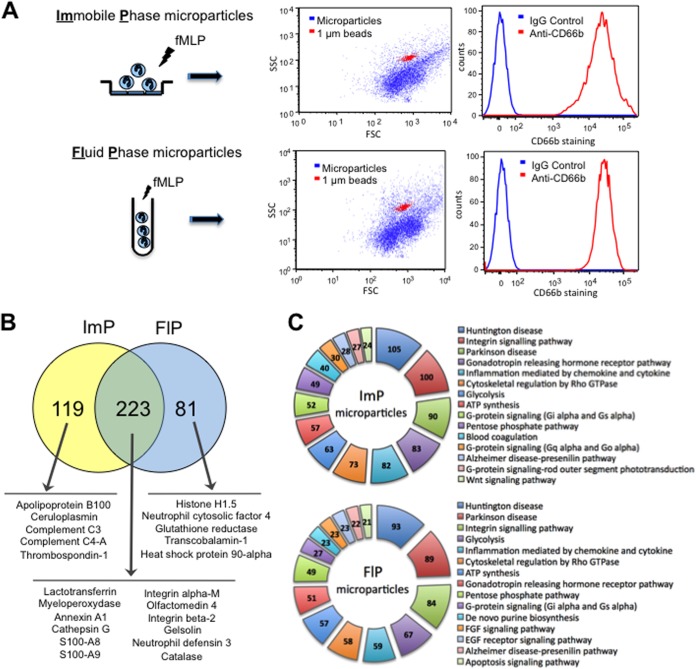
**Differential stimulation of neutrophils yields microparticles with a distinct proteome.** *A*, The physical properties of microparticles - obtained from neutrophils after stimulation in fluid-phase (FlP; in suspension) or immobilized-phase (ImP; post adhesion to a HUVEC monolayer - were assessed employing the forward and side scatter parameters on the dot-plot generated by flow-cytometric analysis. The origin from the neutrophil was ascertained by staining the microparticles by anti-CD66b staining. *B*, Venn diagrams representing the proteomic content identified in each of the neutrophil microparticle subsets as determined using tandem LC-MS-MS. *C*, Ingenuity Pathway Analysis software was used to highlight the top 15 functions of the various proteins expressed in the distinct microparticle subsets as illustrated. In all cases results are representative of four distinct analyses.

Neutrophil microparticles can induce distinct effects on recipient cells by promoting either activating/pro-inflammatory (24, 25) or inhibiting/anti-inflammatory effects (12, 24–26, 36). Here we investigated whether these multiple actions of neutrophil microparticles resulted from a distinct proteomic profile. Freshly prepared neutrophils were incubated either in suspension (FlP) or over a HUVEC monolayer (ImP) for 20 min prior to stimulation. Flow cytometry was employed to confirm the presence of microparticle in our preparations, staining against the CD66b antigen (11), and calibrating against 1-μm beads (Fig. 1*A*). Over 95% of the counts were CD66b^+^ in both preparations (Fig. 1*B*) with no detectible contribution of platelet, monocytes or endothelial microparticles in these preparations (supplemental Fig. S1) as determined by flow-cytometirc staining for CD62P, CD41 (platelet), CD14 (monocyte), and CD54 (endothelial) positive microparticles (supplemental Fig. S1). Adherent neutrophils were found to produce a larger number of microparticles (ImP microparticles) than neutrophils stimulated in suspension (FlP microparticles). Of note there were no significant differences in overall size as determined by the forward and side scatter parameters in flow cytometry between these two subsets (Fig. 1*A*). These results suggest that the microparticle populations obtained under these conditions displayed similar physical characteristics with no detectible contribution by other cell types.

##### Proteomics Analysis of ImP and FlP Microparticles

Tandem gel-LC-MS-MS proteomic analysis was performed on two distinct microparticle preparations from two separate donors. Each of the microparticle preparations was then assayed in duplicate to account for any intrarun variation in the analysis. In this proteomic analysis we identified 342 proteins in the ImP microparticles and 304 proteins in the FlP microparticles. Protein expression, as determined by spectral counting, in each of the microparticle subsets was found to be similar between the two microparticle preparations suggesting minor intra donor variation (Table I). In addition spectral counts were also found to be similar in each of the duplicate runs performed on the microparticle preparations. Around 30% of the proteins were uniquely expressed in one of the microparticle subsets (Fig. 1*B*), suggesting that culture conditions might influence the way microparticles are generated from the same cell type. Table I lists the most abundant 20 proteins in each microparticle subset and a full list can be found in supplemental Table S1. The identified peptides are detailed in supplemental Table S2. Among the most abundant proteins contained in both microparticles populations (223), molecules such as myeloperoxidase, ANXA1, cathepsin G, or S100-A8 (Fig. 1*B*) are typical for this leukocyte type.

**Table I:** Most abundant proteins identified in ImP and FlP microparticles subsets

Sample	Protein name	UNIProt ID	Molecular weight (kDa)	*p* value (t-test ImP *vs* FlP)	AVG Spectral counts	S.E. Spectral counts
*ImP Microparticles*						
	Lactotransferrin	TRFL	78	0.027	829.3	83.2
	Alpha-2-macroglobulin	A2MG	163	0.0000014	723.3	49.1
	Myeloperoxidase	PERM	84	0.081	440.3	35.6
	Ig mu chain C region	IGHM	49	0.00019	317.3	33.7
	Serum albumin	ALBU	69	0.0037	310.0	15.7
	Haptoglobin	HPT	45	0.0002	203.8	20.8
	Integrin alpha-M	ITAM	127	0.27	202.5	12.2
	Serotransferrin	TRFE	77	0.00016	161.3	19.7
	Annexin A6	ANXA6	76	0.48	131.5	8.2
	Apolipoprotein B-100	APOB	516	0.0000044	127.8	12.8
	Hemoglobin subunit beta	HBB	16	0.74	112.8	13.8
	Ig gamma-1 chain C region	IGHG1	36	0.0000072	108.8	7.5
	Histone H4	H4	11	0.049	107.8	23.3
	Hemoglobin subunit alpha	HBA	15	0.55	107.0	5.6
	Complement C3	CO3	187	0.00021	97.0	15.3
	Actin, cytoplasmic 1	ACTB	42	0.019	95.0	7.2
	Annexin A1	ANXA1	39	0.19	93.3	3.0
	Integrin beta-2	ITB2	85	0.65	91.3	6.6
	Myosin-9	MYH9	227	0.49	87.0	16.7
	Ceruloplasmin	CERU	122	0.0004	85.3	14.6
*FlP Microparticles*						
	Lactotransferrin	TRFL	78	0.027	852.3	26.3
	Myeloperoxidase	PERM	84	0.081	469.3	44.2
	Integrin alpha-M	ITAM	127	0.27	172.8	16.2
	Serum albumin	ALBU	69	0.0037	149.8	5.3
	Histone H4	H4	11	0.049	123.3	6.4
	Annexin A6	ANXA6	76	0.48	107.3	13.9
	Actin, cytoplasmic 1	ACTB	42	0.019	104.8	15.1
	Myosin-9	MYH9	227	0.49	103.3	47.7
	Annexin A1	ANXA1	39	0.19	101.3	19.4
	Catalase	CATA	60	0.0023	98.8	12.9
	Protein S100-A8	S10A8	11	0.0021	88.5	3.8
	Plastin-2	PLSL	70	0.034	88.5	29.5
	Hemoglobin subunit beta	HBB	16	0.74	88.0	7.1
	Matrix metalloproteinase-9	MMP9	78	0.056	78.3	5.3
	Annexin A3	ANXA3	36	0.002	76.3	4.5
	Hemoglobin subunit alpha	HBA	15	0.55	75.0	5.1
	Annexin A11	ANX11	54	0.38	73.3	15.0
	Integrin beta-2	ITB2	85	0.65	69.8	9.0
	Heat shock 70 kDa protein 1	HSP71	70	0.031	68.5	15.3
	Glyceraldehyde-3-phosphate dehydrogenase	G3P	36	0.0046	62.0	5.6

A functional analysis of canonical pathways was performed on each microparticle subset (Fig. 1*C*). The pathways significantly associated with both subsets were mainly immune-related pathways: integrin signaling, inflammation mediated by cytokines and chemokines or EGF and FGF signaling pathways. Despite the important difference in their protein profile, this type of functional analysis did not reveal any evident difference between both microparticle subsets, possibly because it only accounts for the number of proteins involved in a given pathway regardless of expression level and specific role in the pathway—activator/repressor—and hence it has limited utility. However in both cases the functional analysis suggests that both microparticles subsets might have immune-modulatory functions.

##### Determining the Expression Profiles of a Select Group of Identified Proteins in the Two Microparticle Subsets

**Fig. 2.**
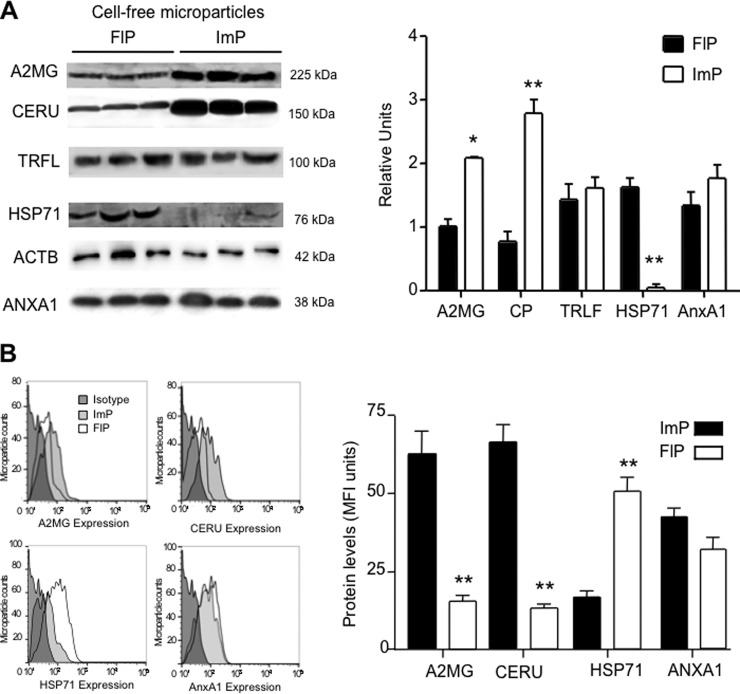
**Stimulus-dependent protein expression in neutrophil microparticles.** *A*, Western blotting selecting proteins that were either predominantly (alpha-2-macroglobulin, A2MG; ceruloplasmin, CERU) or uniquely (heat shock 70kDa protein 1, HSP71) expressed in one of the microparticle subsets, or expressed in both sets (Annexin A1, ANXA1; Lactoferrin, TRLF and β-actin, ACTB). ACTB was used as a loading control for densitometry analysis. and (*B*) flow cytometric analyses of a select group of proteins identified in the proteomic screen (see Methods for details). Results are mean ± S.E. of *n* = 3–4 distinct microparticle preparations.

We next assessed the abundance of a select group of proteins identified by the proteomic screen in the two microparticle subsets by Western blotting. To better evaluate protein expression in each of the two-microparticle subsets we loaded equal number of microparticles for each of the subsets. Here we found that that alpha-2-macroglobulin (A2MG) and ceruloplasmin (CERU) were enriched in the ImP microparticles, while heat shock 70kDa protein 1 (HSP71) was elevated in FlP microparticles (Fig. 2*A*). Annexin A1 (ANXA1), Lactoferrin (TRLF), and β-actin (ACTB) were expressed at equal levels in the two microparticles subsets. Densitometic analysis employing ACTB as a loading control further corroborated the relative protein distribution in these two microparticle subsets (Fig. 2*A*). Flow cytometric assessment of this select group of proteins demonstrated that A2MG and CERU were present on the surface of ImP microparticles, HSP71 levels were more abundant on FlP microparticles whereas ANXA1 was equally expressed on the surface on both microparticle subtypes (Fig. 2*B*). These results corroborate the finding that distinct neutrophil stimulation yields microparticles with a characteristic proteomic profile.

##### Are ImP and FlP Microparticles Produced in Human Inflammation?

To determine the translational value of our observations to the *in vivo* clinical scenario, we measured expression of a select number of proteins under two distinct inflammatory conditions. The exudates from experimental cantharidin-elicited skin blister model, which leads to formation of a highly neutrophilic response and plasma from patients suffering from severe sepsis or septic shock resulting from community acquired pneumonia (CAP), these were compared with plasma neutrophil microparticles obtained from healthy volunteers (Table II).

**Table II:** Microparticle counts and demographic information on patients samples

Sample	PMN counts (ml^−1^)	Exudate volume (ml)	Age (years)	Sex	CD66b^+^ microparticles (%)	Total microparticle count (ml^−1^)
Healthy volunteer plasma	6.70 × 10^6^ ± 1.60 × 10^6^	Not applicable	30.0 ± 0.5	9 m/7F	6.5 ± 4.5	29567 ± 2071
Blister exudate	0.62 × 10^6^ ± 0.22 × 10^6^	1.15 ± 0.65	31.3 ± 0.4	6 m/4F	39.9 ± 11.5	63367 ± 1423
CAP sepsis plasma	10.27 × 10^6^ ± 0.51 × 10^6^	Not applicable	58.8 ± 1.6	2 m/8F	12.3 ± 5.2	40093 ± 1670

**Fig. 3.**
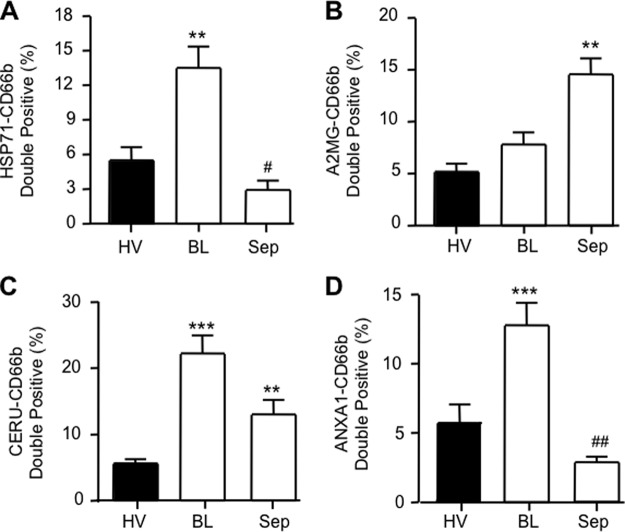
**Microparticle heterogeneity in human samples.** Expression levels of A2MG, CERU, HSP71, ANXA1 in CD66b^+^ microparticles in plasma obtained from healthy volunteers (HV) or septic patients (Sep), as well as in exudates obtained from cantharidin-elicited skin blisters (BL). Data are mean ± S.E. of 10–16 samples tested in duplicate. *p < 0.05, ** *p* < 0.01 *versus* CT group; ++ *p* < 0.01 *versus* respective BL group.

Flow-cytometric analysis for expression of A2MG, CERU, HSP71 and ANXA1 on the surface of neutrophil microparticles demonstrated that CD66b^+^ microparticles harvested from blister exudates contained higher levels of HSP71 (13.8 ± 0.5% *versus* 5.7 ± 0.2%), CERU (22.2 ± 0.7% *versus* 5.6 ± 0.1%) and ANXA1 (12.8 ± 0.4% *versus* 5.7 ± 0.2%) when compared with healthy volunteer plasma neutrophil microparticles (Fig. 3). On the other hand, elevated levels of A2MG (14.6 ± 0.3% *versus* 5.2 ± 0.1%) and CERU (13.4 ± 0.5% *versus* 5.6 ± 0.1%) were observed in plasma neutrophil microparticles from CAP patients, with a significant reduction in HSP71 and ANXA1 expression in relation to blister-derived and healthy volunteer plasma microparticles (Fig. 3). These results confirm that selected proteins identified in our proteomic profiling are regulated *in vivo* and can indeed be expressed by human neutrophil microparticles under both homeostatic conditions and during local or systemic inflammatory responses.

##### ImP and FlP are Also Effectors of Inflammation

**Fig. 4.**
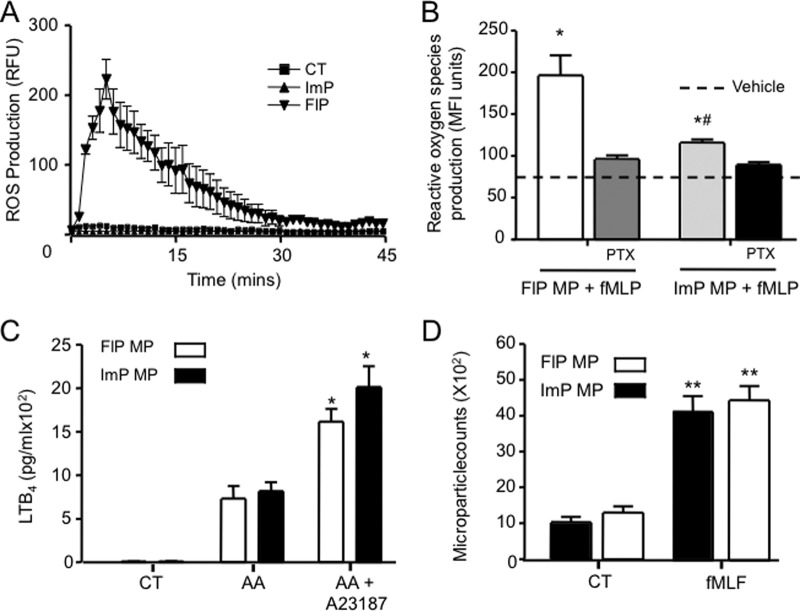
**Neutrophil microparticles respond to external stimuli.** *A*, The ability of FlP and ImP microparticles (∼6 × 10^4^) to produce ROS as assessed with lucigenin, following stimulation with fMLF (1 μm). *B*, The ability of FlP and ImP microparticles to produce ROS in a receptor dependent fashion was assessed after loading the microparticles with DCFDA and stimulation with 1 μm fMLF, with or without pertussis toxin (PTX,1 μg/ml). *C*, LTB_4_ release from microparticles (∼6 × 10^4^) after incubation with arachidonic acid (AA, 100 nm), or in the presence of AA, (100 nm) and the calcium ionophore A23187 (10 nm, 15 min, 37 °C. *D*, Microparticle chemotaxis toward fMLF (1 μm) after 1 h at 37 °C. Microparticle counts in the chemtoaxis chamber of a ChemoTx ® were determined by flow cytometry (calibration conducted with 1 μm beads) as a function of AnxAV positive events. Results are mean ± S.E. *n* = 4 distinct microparticle preparations. *p < 0.05, ** *p* < 0.01 *versus* CT group; #*p* < 0.05 *versus* FIP microparticles.

We next investigated whether these microstructures were mere vehicles carrying proteins from source to sink, or whether they could act as effectors in their own respect. Analysis of the proteome highlighted the presence of various components of the NADPH oxidase complex in the FlP microparticles including NCF2 (p67phox) and NCF4 (p40phox; supplemental Table S1 and Table II). We also found evidence for the presence of CY24B (NOX2; supplemental Tables S1 and S2) and NCF1 (p47phox; supplemental Fig. S2*A*; these were not included in the list of identified proteins because they did not satisfy in full the identification criteria as outlined in the methods section). These findings prompted us to investigate whether FlP microparticles produced reactive oxygen species (ROS) upon direct stimulation. Addition of 1 μm fMLF to FlP microparticles led to ROS production, monitored as increase in fluorescence, an action that was not shared by ImP microparticles, (Fig. 4*A*). Addition of pertussis toxin blocked ROS production in response to fMLF (Fig. 4*B*) indicating that this was a receptor-mediated response.

The proteomic screen also identified leukotriene A_4_ hydrolase (LKHA4) in the proteome of FlP microparticles (supplemental Tables S1 and S2) and indicated the presence of 5-lipoxygenase (5-LOX) in these microparticles (supplemental Fig. S1*B*). Thus we investigated whether the microparticle subsets identified herein could generate LTB_4_ on stimulation. Of note, both microparticle subsets produced LTB_4_ in the presence of arachidonic acid (732 ± 82 pg/ml and 820 ± 58 pg/ml for FlP and ImP microparticles respectively), a process that was enhanced when microparticles were incubated in presence of the calcium ionophore A23187 (Fig. 4C).

Because the proteomic screen demonstrated presence of 37 cytoskeletal related proteins in ImP microparticles and 40 in FlP microparticles including Tubulin Beta-5 Chain, β-Actin (ACTB), and Myosin 6 (supplemental Tables S1 and S2), we next investigated whether these microparticles could migrate down a chemotactic gradient. Incubation of microparticles in the presence of fMLF, a potent chemoattractant, led to a significant migration in the collecting chamber of the chemotaxis plate when compared with microparticles incubated in the presence of buffer alone (Fig. 4*D*). Together these results suggest that microparticles may move to specific tissue sites and exert independent effector functions.

##### Neutrophil Microparticles Elicit Distinct Gene Expression Profiles in Endothelial Cells

Several mechanisms have been reported to explain how microparticles exert their biological actions and how they influence cellular processes (see Introduction). They can interact with surface molecules on a target cell, they can transfer their contents to a target cell by fusion or phagocytosis or they can directly produce ROS, as we showed earlier. Here we next investigated a novel mechanism that is whether microparticles actively modulated the gene expression pattern of target cells producing longer-lasting effects rather than just a passive transfer of their contents. On these bases, we set out to explore whether ImP and FlP microparticles subsets could also modify the gene expression profile of endothelial cells.

**Fig. 5.**
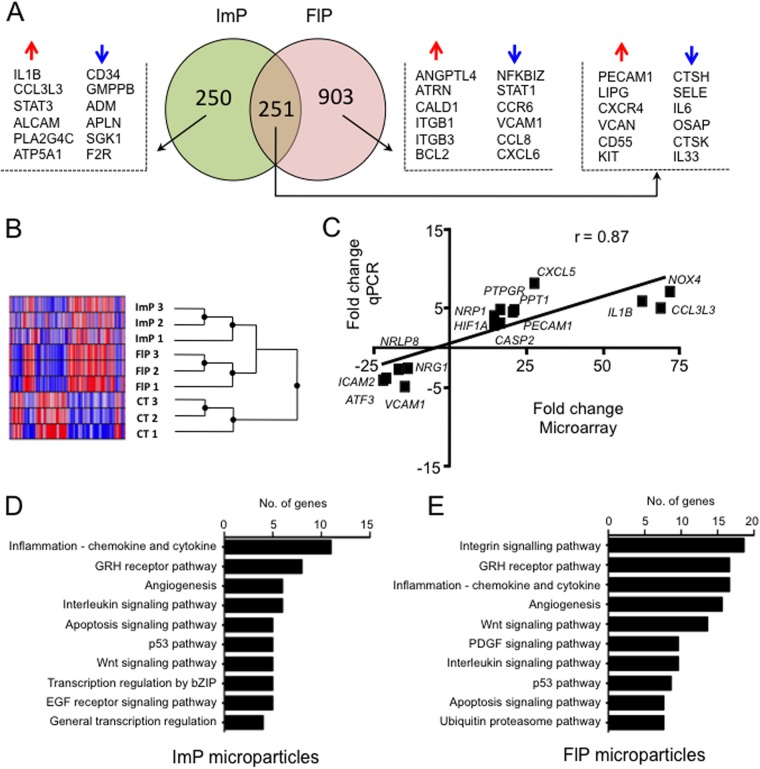
**Neutrophil microparticles modify endothelial cell gene expression profile.** HUVEC were incubated with vehicle, FlP or ImP microparticles (∼8 × 10^5^) for 6 h at 37 °C. After cell harvest and RNA extraction, gene expression profile was assessed using Illumina HT12v4 microarrays. *A*, Venn diagram showing the number of significantly (*p* < 0.05) regulated genes in the ImP and FlP microparticles treated *versus* vehicle cells (PBS). Selected genes are shown. *B*, Heatmap generated with the individual replicates showing intersample similarity. *C*, Real time-PCR validation of 15 distinct genes identified in the microarray analysis, showing very high correlation between the two techniques. Results are cumulative from three separate experiments with distinct microparticle and HUVEC preparations. (*D*, *E*) Functional analysis showing the top 15 pathways associated with ImP and FlP microparticles treated HUVEC respectively.

As shown in Fig. 5*A*, microparticles had a significant impact on the gene expression pattern of HUVEC cells: 501 and 1154 genes were significantly modulated in endothelial cells when co-incubated with ImP and FlP microparticles, respectively (see also Table III and supplemental Table S3). A total of 251 genes were significantly altered in both conditions whereas a substantial number of genes were affected only by one of the microparticle subsets. The triplicates used in our study showed very high consistency (Fig. 5*B*) and a real time-PCR confirmation of selected genes showed an 87% correlation between microarray and PCR data, validating the microarrays analysis. In line with the results obtained for the microparticle proteomes, a functional analysis of canonical pathways did not reveal major differences between both microparticles treatments, however it indicated that the genes affected are mainly related to immune responses, such as integrins and cytokines as well as angiogenesis and apoptosis.

**Table III:** Genes regulated in HUVEC treated with ImP or FlP microparticles. The top 10 up- and down-regulated genes are shown for each microparticle subset

Sample	Probe ID	Gene name	Symbol	Fold change	p value
*ImP Microparticles*					
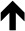	ILMN_1794782	ATP-binding cassette, sub-family G, member 1	ABCG1	70.6	0.0000
	ILMN_2355033	KIAA1147	KIAA1147	45.6	0.0000
	ILMN_1658094	Zinc finger protein 365	ZNF365	42.5	0.0001
	ILMN_1768534	Basic helix-loop-helix family, member e40	BHLHB2	39.1	0.0001
	ILMN_1775501	Interleukin 1, beta	IL1B	39.0	0.0001
	ILMN_2189027	Lipase, endothelial	LIPG	38.9	0.0001
	ILMN_1689037	Lipase, endothelial	LIPG	38.1	0.0002
	ILMN_3253456	Fibronectin type III domain containing 3B	FNDC3B	38.1	0.0002
	ILMN_1801584	Chemokine (C-X-C motif) receptor 4	CXCR4	36.8	0.0002
	ILMN_1699695	Tumor necrosis factor receptor superfamily, member 21	TNFRSF21	36.1	0.0002
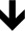	ILMN_1774207	Angiopoietin 2	ANGPT2	−36.2	0.0002
	ILMN_1747759	WD repeat and SOCS box containing 1	WSB1	−36.7	0.0002
	ILMN_2117323	Phosphatidylinositol-4-phosphate 3-kinase, catalytic subunit type 2 beta	PIK3C2B	−40.0	0.0001
	ILMN_1758895	Cathepsin K	CTSK	−40.5	0.0001
	ILMN_2079098	INTS3 and NABP interacting protein	C9orf80	−46.8	0.0000
	ILMN_1773337	Dickkopf 1 homolog (Xenopus laevis)	DKK1	−46.8	0.0000
	ILMN_1844593	—	–	−52.4	0.0000
	ILMN_1739393	Selectin E	SELE	−53.8	0.0000
	ILMN_1699651	Interleukin 6	IL6	−54.3	0.0000
	ILMN_1793025	Mitochondria-localized glutamic acid-rich protein	OSAP	−64.3	0.0000
*FlP Microparticles*					
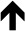	ILMN_1794782	ATP-binding cassette, sub-family G, member 1	ABCG1	138.3	0.0000
	ILMN_2189027	Lipase, endothelial	LIPG	133.0	0.0000
	ILMN_1658494	Chromosome 13 open reading frame 15	C13orf15	120.3	0.0000
	ILMN_1741847	Matrix metallopeptidase 10 (stromelysin 2)	MMP10	117.5	0.0000
	ILMN_2355033	KIAA1147	KIAA1147	117.1	0.0000
	ILMN_1721138	GrpE-like 2, mitochondrial (E. coli)	GRPEL2	116.9	0.0000
	ILMN_1740407	Chondroitin sulfate synthase 3	CHSY3	105.6	0.0000
	ILMN_1800540	CD55 molecule, decay accelerating factor for complement	CD55	104.8	0.0000
	ILMN_2309156	Prostate transmembrane protein, androgen induced 1	PMEPA1	93.0	0.0000
	ILMN_1790160	v-kit Hardy-Zuckerman 4 feline sarcoma viral oncogene homolog	KIT	91.6	0.0000
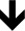	ILMN_1798654	Minichromosome maintenance complex component 6	MCM6	−70.1	0.0000
	ILMN_1754920	Chromosome 6 open reading frame 58	C6orf58	−73.9	0.0000
	ILMN_2398926	Chromosome 17 open reading frame 58	C17orf58	−76.8	0.0000
	ILMN_1739393	Selectin E	SELE	−79.1	0.0000
	ILMN_1793025	Mitochondria-localized glutamic acid-rich protein	OSAP	−82.6	0.0000
	ILMN_1742332	Potassium channel tetramerisation domain containing 12	KCTD12	−87.1	0.0000
	ILMN_1808789	Myosin VC	MYO5C	−90.9	0.0000
	ILMN_1719695	Nuclear factor of kappa light polypeptide gene enhancer in B-cells inhibitor, zeta	NFKBIZ	−111.8	0.0000
	ILMN_1668125	Myosin VIIA and Rab interacting protein	MYRIP	−157.3	0.0000
	ILMN_1810810	Eukaryotic translation elongation factor 1 alpha 1	EEF1A1	−371.3	0.0000

A detailed study of the genes altered in each subset uncovered important differences: the up-regulation of pro-inflammatory genes by ImP microparticles, such as IL1β, CCL3L1, or STAT3, together with the down-regulation in FlP of genes such as STAT1, NFKBIZ, CCL8, or CXCL6 suggest a pro-inflammatory phenotype for ImP microparticles and an anti-inflammatory phenotype for FlP microparticles. In addition, incubation of ImP microparticles with HUVEC led to down-regulation of genes with important roles in vascular function including adrenomedullin (ADM), a protective factor for blood vessels, apelin (APNL) which participates in the control of blood pressure, or the enzyme serum and glucocorticoid regulated kinase 1 (SGK1), which regulates endothelial cells apoptosis. On the contrary, protective factors were up-regulated by the FlP subset, including ANGPTL4 that acts as an endothelial cell survival factor, and CD55 (an anti-inflammatory receptor for complement).

Finally, we performed an *in silico* comparison of our gene expression profiles, with those produced by more than 1300 drugs contained in The Connectivity Map (CMap) database. We found that the top 10 drugs with the highest score, that is, with a similar gene expression profile in the FlP microparticles (Table IV), included an immunosuppressant (phenanthiridinone), a cytoprotective agent (16,16-dimethylprostaglandin E_2_), and the anti-inflammatory drugs SC-560 and NS-398 (selective COX-1 and COX-2 inhibitors, respectively), providing further evidence of the potential anti-inflammatory nature of the FlP microparticles. On the other hand, although not on the top 10 drugs shown in Table IV, several antimicrobial/antifungal drugs were positively associated with ImP microparticles-treated HUVEC, such as ikarugamycin (score 0.371), josamycin (0.366), thiamphenicol (0.353), furazolidone (0.302), vancomycin (0.281), or amphotericin B (0.279).

**Table IV:** Connectivity map drugs associated (positive score) with ImP and FlP microparticles treated HUVEC. The top 10 drugs according to score are shown

Sample	Drug	Activity	Score	n	*p* value	% non-null
*ImP Microparticles*						
	12,13-EODE (iso-leukotoxin)	Produced by neutrophils during oxidative burst	0.697	1	—	100
	5162773	Unknown	0.676	1	—	100
	(−)-Catechin	Antioxidant	0.654	1	—	100
	Cytochalasin B	Antimicotic	0.555	1	—	100
	Topiramate	Anticonvulsant	0.545	1	—	100
	5151227	Unknown	0.535	1	—	100
	Tomelukast	Leukotriene D_4_ antagonist	0.521	1	—	100
	5186324	Unknown	0.516	1	—	100
	Pralidoxime	Cholinesterase reactivator	0.477	4	0.03617	75
	5213008	Unknown	0.465	1	—	100
*FlP Microparticles*						
	Phenanthridinone	Immunosuppressant	0.644	1	—	100
	Topiramate	Anticonvulsant	0.636	1	—	100
	Demecolcine	Anticancer	0.574	1	—	100
	2-deoxy-d-glucose	Glycolysis inhibitor	0.550	1	—	100
	16,16-dimethylprostaglandin E2	Cytoprotective	0.523	3	0.00176	100
	Naltrexone	Opioid antagonist	0.482	5	0.00192	80
	SC-560	Selective COX-1 inhibitor	0.473	3	0.08261	66
	NS-398	Selective COX-2 inhibitor	0.448	3	0.09723	66
	5279552	Unknown	0.438	2	0.50195	50
	Mevalolactone	Anti-oxidant	0.436	3	0.08749	66

## DISCUSSION

We report herein that neutrophil stimulation can lead to the generation of heterogeneous microparticles characterized by distinct proteomes. This difference in protein composition confers characteristic functional abilities to microparticles (*e.g.* the ability of FlP microparticles to produce ROS). In addition, microparticles produced by the same cell type in response to different stimuli (*i.e.* in adhesion *versus* in suspension) induced discrete gene expression profile once added to recipient cells.

Since their discovery there has been considerable progress in appreciating that cell-derived microparticles may play an active role in homeostasis and disease (3, 37–39). Recent evidence suggests that microparticles exert potent actions in cell-to-cell communication in both normal physiology and disease. To date, most studies have focused on the cellular origin of microparticles during disease (for a comprehensive review on endothelial microparticles see (14)) whereas only limited information is available on the protein composition of these microstructures, as well as their potential downstream actions once released into the surrounding milieu by the parent cells. In line with previous studies conducted with a human monocytic cell line and endothelial cells (9, 40) we provide evidence for heterogeneity in neutrophil microparticles produced in response to discrete stimuli. In line with our findings Timár and colleagues recently demonstrated that only in response to opsonized *S. aureus* neutrophils produced microparticles endowed with antibacterial properties (36).

One of the initial steps in the activation cascade of a blood-borne neutrophil that leads to its recruitment to the site of inflammation is interaction with venular endothelium to begin the process of extravasation (41, 42). Indeed, indication for microparticle generation during neutrophil migration across the endothelial wall has recently been provided (23), further underscoring the importance of establishing the content of these microparticles. Furthermore, evidence from clinical studies in a number of diseases suggests that neutrophil microparticles may be useful biomarkers in a number of inflammatory pathologies. For example, in conditions such as vasculitis and severe injury, plasma CD66b^+^ microparticle counts are elevated (43, 44). However, it is difficult to understand the potential influence that such microparticles could be exerting without first appreciating their composition.

For all these reasons, herein we focused on the neutrophil microparticle proteome employing two distinct culture conditions with freshly prepared neutrophils. This yielded microparticles with similar physical properties, as deduced by flow cytometry. Proteomic analysis of these microparticle subsets demonstrated that they possessed distinct proteomic profiles with about 30% of the total proteins identified for the two subsets being uniquely expressed in one of the microparticle subsets. Protein expression profiles obtained by Western blotting for a select group of proteins identified during the proteomic screen corroborated the results obtained by LC-MS-MS, both with respect to their identity and distribution between the two microparticle groups. The detection of these proteins was highly reproducible in view of the four samples analyzed by proteomics (see Methods) but also the several assays of Western blotting performed: some of the blots are reported in here, but have been reproduced (*e.g.* for ANXA1) in over 12 distinct preparations of neutrophil microparticles (data not shown). In addition we propose that both ANXA1 and TRLF may be employed as sensitive loading controls in these microparticle subsets because they were equally expressed in both microparticle subsets and found to be at higher levels than β-actin. These findings extend those made in our initial study that focused on ANXA1^+^ microparticles (detected in adherent—but not resting—conditions; see ref (12).), that we found to mediate, at least in part, the acute nongenomic anti-inflammatory properties exerted by this neutrophil microparticle subset (9). The observation that neutrophils have the ability to respond to a specific stimulus by producing microparticles loaded with a distinct proteomic profile supports the notion that microparticle production is a regulated process and that they might be endowed with very discrete functions, as shown herein and discussed below. The hypothesis is further supported by the finding that neutrophil microparticles also exert antibacterial properties (36). It is noteworthy that proteins known to be expressed on neutrophil microparticles such as l-selectin (CD62L) and CD66b were not identified in our proteomic screen. This may be due to a low expression of these proteins in the two microparticle subsets investigated herein when compared with the identified proteins. In addition these proteins are also known to undergo glycosylation, a modification that not only influences their ability to migrate in the SDS-gel but also decreases the probability for mass spectral identification.

To better appreciate the translational potential of our findings we next investigated whether the proteins identified herein were also expressed on neutrophil microparticles obtained from three distinct human settings. Here we assessed microparticles from skin blister exudates since this characterized by a highly neutrophilic inflammation (28), predicting it might reflect the FlP microparticle phenotype. We also profiled plasma microparticles from patients suffering from sepsis, tested as a sample likely to yield ImP-like microparticles, in view of the central role of endothelial activation in this disease (45). The data obtained satisfied to a large extent this hypothesis, stressing the importance of thorough profiling of neutrophil microparticles especially under disease conditions. For instance, elevated expression of the anti-inflammatory proteins ANXA1 or HSP71 in neutrophil microparticles may be indicative of a nonpathogenic neutrophil activation, as observed in microparticles obtained from skin blister exudates, a self-resolving inflammatory model. It should be noted that microparticles bearing at least one of these proteins, ANXA1, can indeed elicit rapid nongenomic anti-inflammatory and homeostatic effects (12). On the other hand, microparticles purified from septic patients had dramatically elevated A2MG levels and a concomitant reduction in HSP71 and ANXA1 levels, reflecting a distinct, neutrophil activation profile with the role for these microparticles in the pathology of sepsis remaining of interest and will need to be addressed in future studies.

Next we determined whether microparticles could elicit distinct biological responses, investigating specific actions in relation to the proteomic results. In this context we assessed whether microparticles could be functionally activated to produce ROS (46). These chemical entities play an important role in protection against invading pathogens although uncontrolled ROS production can be detrimental and the various components of this complex are normally brought together to the cell membrane or phagosomal surface only following stimulation (47). Recently, a second role has been ascribed to ROS, distinct from their bactericidal actions, whereby lower levels of ROS induce cytoprotective effects by induction of hypoxia-inducible factor-1α (48). Stimulation of FlP microparticles (but not ImP) with fMLF led ROS production; additionally, ROS levels measured for these microparticles were significantly lower then those produced by an equal number of neutrophils (*n* = 3 experiments; not shown), suggesting that ROS from FlP microparticles might evoke cytoprotective effects, although this will need to be investigated in further studies.

Intriguingly, we also found that microparticles could also move in response to a chemotactic gradient (Fig. 4), in line with the identification of several cytoskeletal proteins in the microparticle proteome (supplemental Table S1). This suggests that microparticles may be able to specifically migrate to inflammatory loci possibly reaching relatively distant target cells to affect downstream inflammatory events. In addition, our results suggest that incubation of neutrophils with fLMF leads to the functional incorporation of formyl peptide receptors (FPR) to microparticles. Engagement of GPCRs by their cognate agonists may lead to receptor internalization where the receptor may either be targeted for degradation or recycled to the cell surface (49). Our finding suggest that FPR receptor may be recycled to the cell surface following agonist binding where in turn it may be incorporated into microparticles. Moreover, these data suggest that receptor signaling in microparticles follows distinct dynamics to those found in the parent cells which will need to be explored in more detailed future studies.

LTB_4_ is a lipid mediator biosynthesized *via* the conversion of arachidonic acid by the actions of two enzymes, 5-lipoxigenase and leukotriene A_4_ hydrolase. LTB_4_ is a potent neutrophil chemo-attractant leading to neutrophil chemotaxis, aggregation, and transmigration across the epithelium and/or endothelium to the site of inflammation. The ability of both microparticle subsets to produce LTB_4_ on stimulation is noteworthy in the context of an orchestrated inflammatory response, suggesting that microparticles can propagate the production of “danger signals.” Equally possible, is that the persistence of these microparticles within the vasculature or a given tissue (*e.g.* rheumatoid arthritis joint) could prove to be detrimental and lead to chronic disease (43, 50).

We concluded the present study by determining if the unique proteomic content of the two microparticle preparations could be sensed, at the genetic level, by recipient cells thus opening the possibility for long-lasting downstream actions of these vesicles. Both microparticle subsets regulated genes that are involved with mitosis, intracellular signaling protein transport and oxidative phosphorylation among others. Of note incubation of FlP with endothelial cells produced the most profound changes with respect to the number of genes differentially expressed and the resultant biological processes affected. These experiments demonstrate that microparticles with different proteomes, albeit generated from the same cell type for the same donor, can elicit profound alterations in gene expression. To better appreciate the functional relevance of this response we used a recently developed tool, the CMap database, that compares the gene expression profiles to those elicited by know drugs. This demonstrated that FlP microparticles induced a gene signature that is reminiscent of molecules with an inhibitory—if not anti-inflammatory, *e.g.* the positive association with COX inhibitors—profile (Table IV). In contrast, incubation of endothelial cells with ImP microparticles induced a gene signature that more resembled the gene print of antibiotics and other drugs able to fight infections. Furthermore assessment of the proteins identified in ImP microparticles supports the notion that these microparticles may exert antibacterial properties (36). These proteins include A2MG and the complement proteins CO3 and CO4A that were mainly or solely identified in ImP microparticles, and the proteins BPI (bactericidal permeability-increasing protein), DEFA3 (neutrophil defensin 3) or OLFM4 (olfactomedin 4), which show higher expression in ImP microparticles.

Neutrophils can orchestrate a variety of responses that go well beyond their classical function of killing bacteria through ROS and proteolytic enzyme release. These cells can produce cytokines and mediators able to influence downstream responses evoked by macrophages, dendritic cells and lymphocytes (see ref (41) for a review). In addition neutrophil microparticles also regulate macrophage and dendritic cell responses in the presence of pro-inflammatory stimuli via the Mer receptor tyrosine kinase (MerTK) and PI3K/Ak pathways (51). Neutrophil microparticles also stimulate the efferocytosis of apoptotic cells (1, 52) and the biosynthesis of pro-resolving mediators by macrophages (52). Recent work has also demonstrated that neutrophil microparticles carry the precursors for the biosynthesis of proresolving mediators (1, 52) that can be donated to macrophages where they are transformed to bioactive mediators (52). On-going work aims at elucidating whether microparticles are important effectors of some of these responses and the gene modulation we report here for HUVEC goes along this exciting possibility.

In conclusion, we report several novel features of neutrophil microparticles. We show that their production is a tightly regulated process with their proteomic content varying greatly depending on the nature of the stimulus. Moreover, we show that these microstructures carry distinct functional properties with an ability to synthesize inflammatory mediators such as LTB_4_ and to respond to the surrounding milieu through ROS production and chemotaxis, opening novel research avenues into effector roles of microparticles in inflammatory responses. In addition, we show that the two microparticle subsets investigated during this study induced profound and distinct changes in gene expression profiles in recipient cells. These observations shed light on an area of biology that, to date, has been very little explored, highlighting the importance of a more systematic analysis of neutrophil microparticles, especially in disease. Such an analysis could provide us with robust biomarkers in disease and, equally important, the ability to determine the efficacy of specific treatment regimes, paving the way for the development of tailor-made medicines.

## Supplementary Material

Supplemental Data

## Footnotes

^1^ The abbreviations used are:

FlP: Fluid-phase Microparticles
A2MG: Alpha-2-macroglobulin
ACTB: beta actin
ANXA1: Annexin A1
CAP: Commonly Acquired Pneumonia
CMap: Connectivity Map
CERU: Ceruloplasmin
HSP71: heat shock 70 kDa protein 1
HUVEC: human umbilical vein endothelial cells
ImP: immobilized-phase microparticles
TRFL: Lactotransferrin.
