# Supplementary material for: Heterogeneity in Neutrophil Microparticles Reveals Distinct Proteome and Functional Properties
Source: Mol Cell Proteomics. 2013 May 8;12(8):2205–19. doi: 10.1074/mcp.M113.028589 (PMC3734580; doi:10.1074/mcp.M113.028589)
Supplement: Supplemental Data [file supp_12_8_2205__index.html]

Heterogeneity in Neutrophil Microparticles Reveals Distinct Proteome and Functional Properties — Heterogeneity in Neutrophil Microparticles — Supplemental Data 

# Heterogeneity in Neutrophil Microparticles Reveals Distinct Proteome and Functional Properties

## Supplemental Data

**Files in this Data Supplement:**

- MCP:2013:028589 Supplementary Figures - Supplementary Figure 1: No evidence for platelet, endothelial and monocyte microparticles in the neutrophil microparticle preparations. Supplementary Figure 2: Evidence for the presence of NCF-1 and 5-lipoxygenase in FlP microparticles.
- Supplemental Table S1
- Supplemental Table S2
- Supplemental Table S3
