# Supplementary material for: Heterogeneity in Neutrophil Microparticles Reveals Distinct Proteome and Functional Properties
Source: Mol Cell Proteomics. 2013 May 8;12(8):2205–19. doi: 10.1074/mcp.M113.028589 (PMC3734580; doi:10.1074/mcp.M113.028589)
Supplement: Supplemental Data [file supp_M113.028589_mcp.M113.028589-1.pdf]

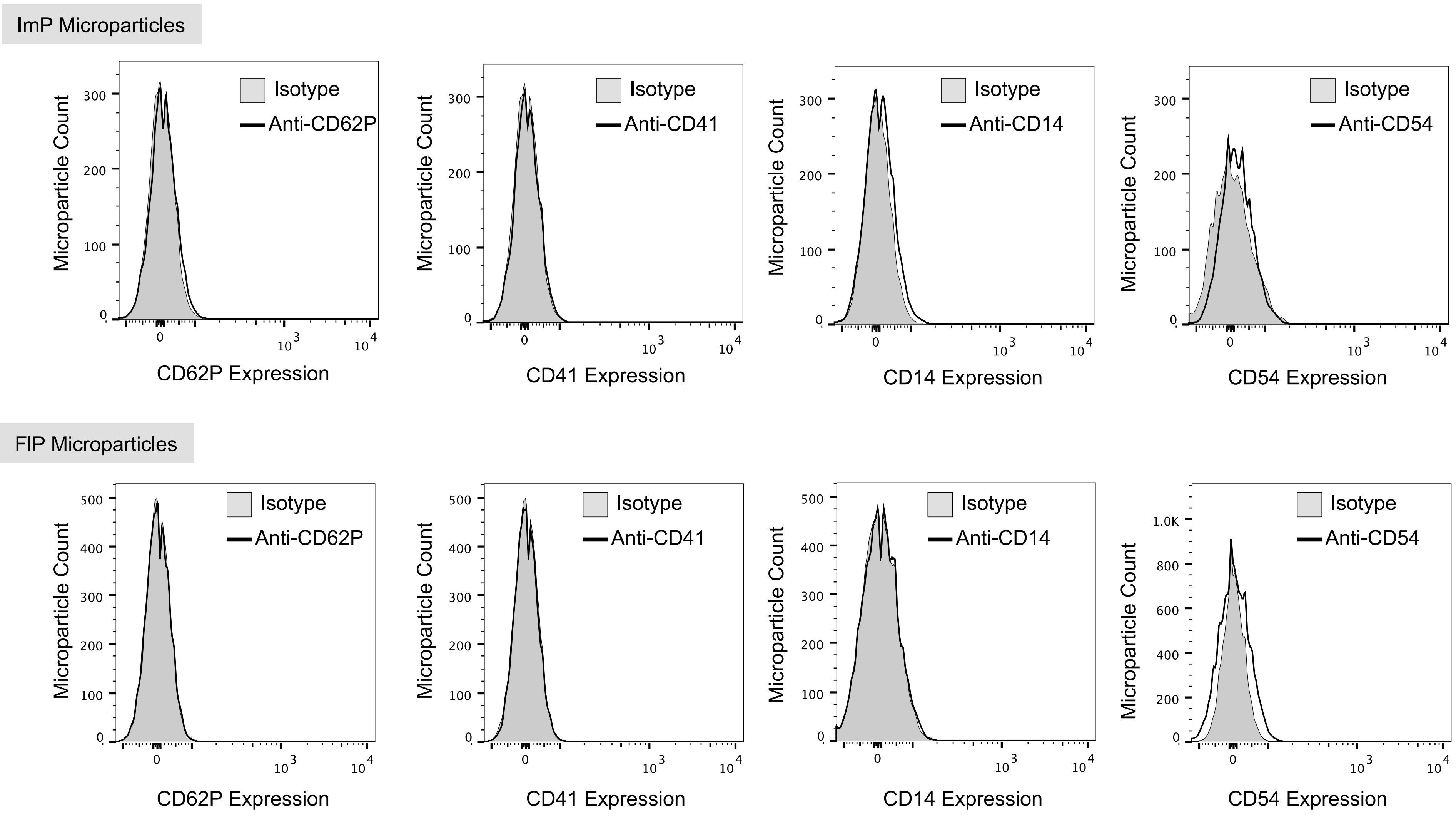

Supplementary Figure 1: No evidence for platelet, endothelial and monocyte microparticles in the neutrophil microparticle preparations. Microparticles were suspended in PBS (containing calcium and magnesium) and incubated with mouse anti human -CD62P, CD41 (platelet microparticle markers), CD54 (endothelial microparticle marker) or anti-CD14 (monocyte microparticle marker) conjugated antibodies. Staining was then assessed using a FACS Canto II and data analysed using FlowJo software**.** Results are representative of n=5 microparticle preparations.

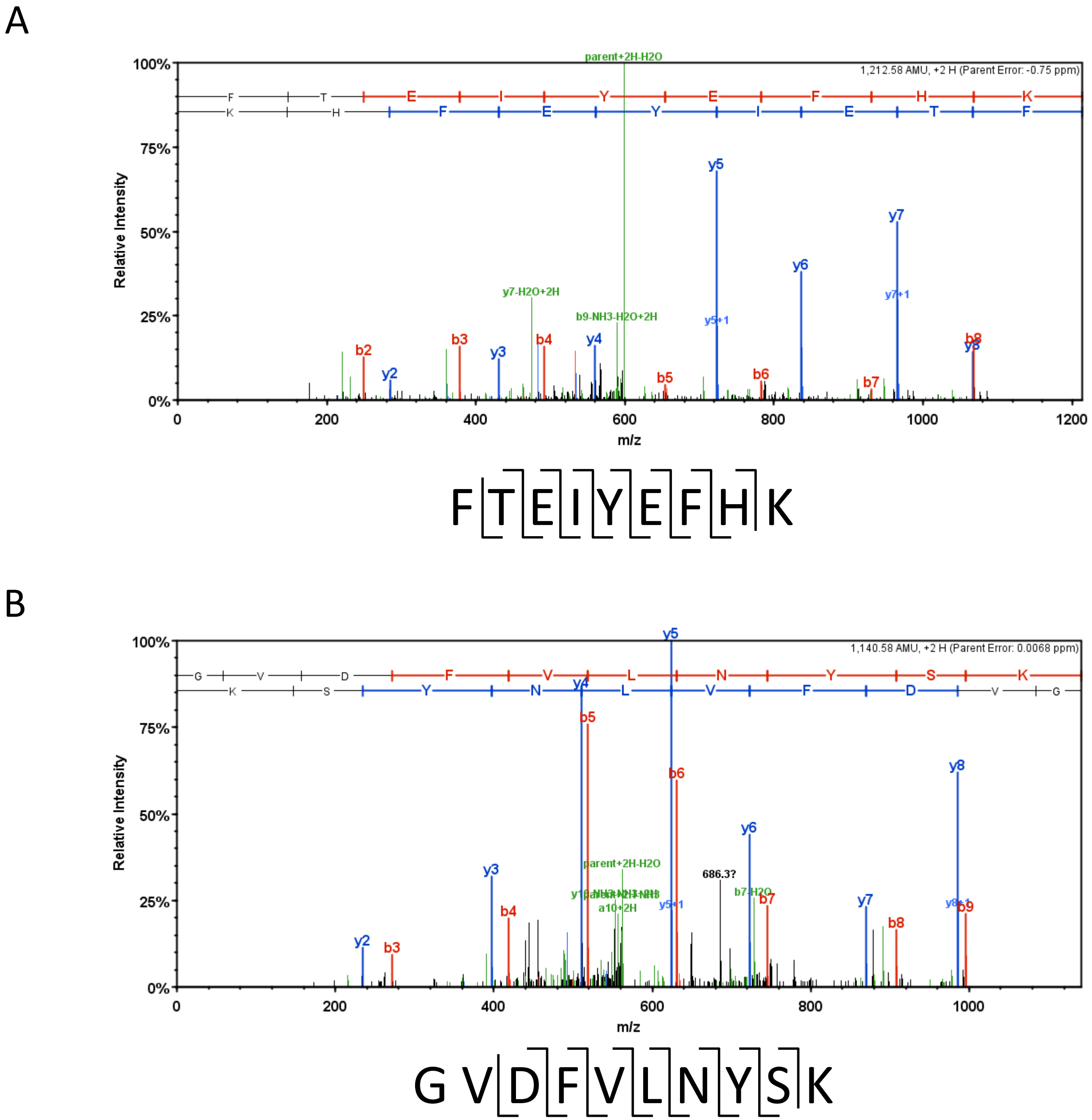

Supplementary Figure 2: Evidence for the presence of NCF-1 and 5-lipoxygenase in FIP microparticles. Mass spectra employed for the identification of (A) NCF-1 and (B) 5 lipoxygenase in FIP microparticles.
